# Evaluating signs of hippocampal neurotoxicity induced by a revisited paradigm of voluntary ethanol consumption in adult male and female Sprague-Dawley rats

**DOI:** 10.1007/s43440-023-00464-6

**Published:** 2023-02-20

**Authors:** Carles Colom-Rocha, Cristian Bis-Humbert, M. Julia García-Fuster

**Affiliations:** 1 IUNICS, University of the Balearic Islands, Cra. de Valldemossa Km 7.5, 07122 Palma, Spain; 2 Health Research Institute of the Balearic Islands (IdISBa), Palma, Spain; 3 Present Address: Psychobiology of Drug Addiction, Neurocentre Magendie, INSERM U1215, Bordeaux, France

**Keywords:** Alcohol voluntary drinking, Sex differences, Hippocampus, Neurogenesis, Rat

## Abstract

**Background:**

Binge alcohol drinking is considered a prominent risk factor for the development of alcohol-use disorders, and could be model in rodents through the standard two-bottle preference choice test. The goal was to recreate an intermittent use of alcohol during 3 consecutive days each week to ascertain its potential impact on hippocampal neurotoxicity (neurogenesis and other neuroplasticity markers), and including sex as a biological variable, given the well-known sex differences in alcohol consumption.

**Methods:**

Ethanol access was granted to adult Sprague–Dawley rats for 3 consecutive days per week, followed by 4 days of withdrawal, during 6 weeks, mimicking the most common pattern of intake in people, drinking over the weekends in an intensive manner. Hippocampal samples were collected to evaluate signs of neurotoxicity.

**Results:**

Female rats consumed significantly more ethanol than males, although intake did not escalate over time. Ethanol preference levels remained below 40% over time and did not differ between sexes. Moderate signs of ethanol neurotoxicity were observed in hippocampus at the level of decreased neuronal progenitors (NeuroD + cells), and these effects were independent of sex. No other signs of neurotoxicity were induced by ethanol voluntary consumption when measured through several key cell fate markers (i.e., FADD, Cyt c, Cdk5, NF-L) by western blot analysis.

**Conclusions:**

Overall, the present results suggest that even though we modeled a situation where no escalation in ethanol intake occurred across time, mild signs of neurotoxicity emerged, suggesting that even the use of ethanol during adulthood in a recreational way could lead to certain brain harm.

**Supplementary Information:**

The online version contains supplementary material available at 10.1007/s43440-023-00464-6.

## Introduction

Alcohol is the legal drug most consumed at world level; in 2020, the last National Survey on Drug Use and Health [1] reported that 50% of people in the USA, ages 12 or older, used alcohol in the past month, and from those, around 44% were classified as binge drinkers and 13% as heavy drinkers. Binge drinking, defined by the National Institute on Alcohol Abuse and Alcoholism (NIAAA) as an enduring period of observable behavioral intoxication that brings blood alcohol concentration to 0.08 g percent (or 80 mg%) or above (e.g., 5 or more drinks for males or 4 or more drinks for females in about 2 h), is a prominent risk factor for later development of alcohol-use disorders (e.g., [2]).

At the preclinical level, binge modeling of ethanol drinking across laboratories has been proven challenging in terms of achieving comparable intoxication levels (as measured by high blood ethanol concentrations), since when provided in a 24-h span, rodents distribute their intake throughout the day. Distinctive paradigms have been characterized for adult rodents utilizing variations of the standard two-bottle preference choice test, which initially allowed continuous unlimited access to one bottle containing an ethanol solution (i.e., [3]; and hundreds of publications afterwards), to then limit its scheduling to better mimic human alcohol intake (i.e., reviewed by [4, 5]). Providing an intermittent access to a bottle with ethanol (i.e., 10–20% solution range) models a progressive increase in intake across time following cycles of exposure and drug removal, with increased preference ratios when ethanol is reintroduced (reviewed by [4, 5]). The most commonly used schedule allows unlimited access to rats for periods of 24 h on alternate days (Mondays, Wednesdays and Fridays), creating multiple 24-h short-withdrawal periods in between ethanol drinking sessions, which have proven to lead to severe consequences (i.e., kindling effect [6, 7] and/or negative affect [8]). Over a period comprising several weeks, rats show a progressive increase in voluntary ethanol intake, modeling the development of an addictive-like phenotype (e.g., [9]; reviewed by [4, 5]). Other intermittent models are based on given access to ethanol only for a limited time each day (e.g., 4 h per day during 4 consecutive days a week [10]). Moreover, additional schedules mimicking the development of addictive-like features, either allowing continuous access to ethanol during several weeks or giving access to ethanol at night when rats are active “drinking in the dark” model (reviewed by [4]), have been extensively utilized leading to multiple publications.

In the present study, however, we decided to revisit the most used paradigm that allows access to ethanol for 3 non-consecutive days a week (e.g., Monday, Wednesday and Friday) with alternate 24-h periods of access and/or withdrawal, to explore the response of a continuous 3-day access (i.e., 72 h of unlimited access) followed by a period of 4 days of withdrawal, mimicking the most common pattern of intake in people, drinking over the weekends in a voluntary manner. This pattern of consumption, although it might not necessarily lead to intoxication and/or alcohol-use disorder, it could still be used as a model in which to study sex-specific neuronal adaptations and/or complications. This is particularly relevant given the well-known differential influence of sex when modeling addiction in animals (e.g., [11]); female rats of many strains and ages (adolescent and/or adult) drink more alcohol than their male counterparts (e.g., [12–14]). Also, ethanol is predominantly harmful in hippocampus (both structural and function-dependent; e.g., [15–18]), and there are well-known sex differences in hippocampal damage following alcohol use (e.g., [19, 20]). Therefore, our goal was to revisit and recreate an intermittent use of alcohol in rats of both sexes, with greater behavioral translation to the pattern observed in humans, in which to ascertain the potential impact on hippocampal neurotoxicity.

In this context, signs of hippocampal neurotoxicity were evaluated at different levels. The negative effects of ethanol on impairing the novel generation of adult neurons in hippocampus are well known (i.e., for its consequences on the different stages of adult neurogenesis see [21]; reviewed by [22]). Prior studies reported sex differences in hippocampal damage (e.g., [19]), including changes in the regulation of adult neurogenesis following ethanol use (e.g., [19, 20]), and other sex-specific differential neurotoxic events (e.g., miRNAs expression [23]; microglia number and reactivity [24]). However, there is still scarce knowledge on the magnitude and/or direction of the events taking place in the hippocampus of female rodents following ethanol exposure. In this context, we compared the potential sex differences in the regulation of the initial stages of adult neurogenesis (i.e., cell proliferation and early neuronal survival). Moreover, to deepen our understanding on the sex-related neurotoxic events that might be taking place in this brain region, we selected some key molecular markers, with certain links to the regulation of adult hippocampal neurogenesis that were previously characterized for adult male rodents in the context of other drugs of abuse. For example, the dysregulation of Fas-Associated protein with Death Domain (FADD), a key cell fate player that could balance cell death vs. plasticity events (reviewed by [25, 26]), paralleled decreased levels of cell proliferation in hippocampus following cocaine exposure [27], suggestive of neurotoxic events in this brain region in male rats. We also studied another marker of the apoptotic pathway (Cytochrome c, Cty c) whose expression was altered by drugs of abuse (i.e., cocaine, MDMA) in hippocampus in conjunction with FADD [28, 29]. Also, Cyclin-dependent kinase-5 (Cdk5) was evaluated since is key in the regulation of neurogenesis [30] and was shown to be modulated in parallel to FADD in hippocampus (see [31]). Finally, the potential structural damage of ethanol was evaluated at the level of neurofilament proteins (e.g., NF-L) as certain drugs of abuse induced neurotoxicity in male rats by decreasing its hippocampal content (e.g., [29]); it was found hyperphosphorylated in hippocampus in response to ethanol toxicity [32], and its circulating levels were altered in heavy drinking in association with lower gray matter thickness [33]. Exploring these molecular events will give us an idea of which pathways and/or events should be further explored in the context of ethanol toxicity in hippocampus for each sex.

## Materials and methods

### Animals

A total of 42 adult Sprague-Dawley rats (21 males and 21 females) bred in the animal facility at the University of the Balearic Islands were used in this study. During the experimental procedures, rats were housed individually in standard cages following a 12-h light/dark schedule (lights on at 8:00AM) in a climate-controlled room (22 °C, 70% humidity) and with unlimited access to a standard diet and water. Rats were given at least 1 week to acclimatize to the housing conditions and the handling prior to the actual experiments, which were performed during the light period (between 10:00 and 12:00 h). All procedures complied with the ARRIVE Guidelines [34], the EU Directive 2010/63/EU and the Spanish Royal Decree 53/2013 for animal experiments, and were approved both by the Local Bioethical Committee (CEEA 100/10/18) and the Regional Government (Exp.: 2018/14/AEXP). All efforts were made to minimize the number of rats used and their suffering. In this context, and to prevent the induction of unnecessary stress in female rats during the experimental procedure, the specific stages of the estrous cycle were not examined. This decision was based on the fact that prior studies suggested that the estrous cycle stage might not be a significant player in the amount of ethanol consumption for female rats (e.g., [35]), but mainly because the cyclicity of females was not part of our research question (see [36]).

### Intermittent access to 20% ethanol in a two-bottle choice test

**Fig. 1:**
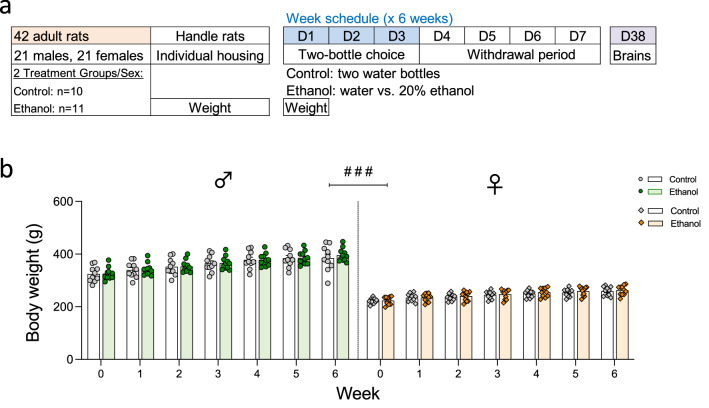
Experimental design. **a** Sprague-Dawley rats were exposed during 6 consecutive weeks to a weekly schedule consisting of a 3-day continued voluntary ethanol access (two-bottle choice: 20% ethanol vs. water; D1–D3) followed by a 4-day withdrawal period (two bottles of water). Rats were killed on the last day of ethanol exposure on week 6 (D38). **b** Changes in body weight across weeks (g). Groups of treatment: Control-male (*n* = 10); Ethanol-male (*n* = 11); Control-female (*n* = 10); Ethanol-female (*n* = 11). Columns represent mean ± SEM of change in body weight (g). Individual symbols are shown for each rat. #*p* < 0.05 when comparing the effect of sex (female vs. male rats; three-way RM ANOVA)

Rats of each sex were randomly allocated into two experimental groups (Control, *n* = 10 and Ethanol, *n* = 11 per sex; see Fig. 1a) and were exposed to two bottles with fluids during 6 consecutive weeks. Each week, rats from the ethanol groups were given unlimited voluntary access to ethanol (20% ethanol vs. water) during 3 consecutive days (i.e., every Tuesdays, Wednesdays and Thursdays) followed by a period of 4 days of withdrawal (all rats had access to 2 bottles of water). Rats from the control groups were always exposed to two bottles of water. Each rat was exposed to a total of 18 ethanol (or control) sessions during the 6-week procedure. The placement of the ethanol bottle was alternated daily to account for side preferences and bottles were weighed every morning during the 3 days of ethanol access. Total fluid intake (sum of both bottles in ml) and the amount of water and/or ethanol consumed (in ml) was recorded on each session day. Ethanol preference was calculated as the amount of ethanol consumed divided by total fluid intake and multiplied by 100 (% values). Weights were monitored weekly (D1 of each week) throughout the course of 6 weeks as detailed in Fig. 1a, and were used to calculate the amount of ethanol consumed (g/kg) daily and cumulative (i.e., ethanol load throughout the whole experimental procedure). Results are expressed as daily consumption, average consumption per week and average consumption during the 18 sessions.

### Immunohistochemistry

Rats were killed by rapid decapitation on the last session/day of week 6 (D38; see Fig. 1a) and the left half-brain was snap-frozen with – 30 °C isopentane (Panreac Química, Barcelona, Spain, cat #143501) and stored at – 80 °C to latter quantify hippocampal neural progenitors by immunohistochemistry at specific periods of regulation (i.e., cell proliferation with Ki-67 and early neuronal survival with NeuroD). Tissue was cryostat-cut (30 µm sections) and slide-mounted throughout the whole hippocampal extent ( – 1.72 to – 6.80 mm from Bregma) in 3 series containing the most anterior, the middle part and the most posterior part of this region as described in detail before [37–39]. For each marker, experiments were performed in 1 slide per rat containing 8 tissue-sections from the middle portion of the hippocampus that were post-fixed in 4% paraformaldehyde (Merck, Darmstadt, Germany, cat #76240). Then, sections were exposed to several steps, such as epitope retrieval in 10% sodium citrate dihydrate (Thermo Fisher Scientific, Waltham, MA, USA, cat #BP327-1) pH 6.0 at 90 °C for 1 h, 0.3% peroxidase solution (Thermo Fisher Scientific, cat #426000010) and BSA blocking (Merck, cat #A7906) before an overnight incubation with rabbit anti-Ki-67 (1:20000) (provided by Drs. Huda Akil and Stanley J. Watson, University of Michigan, MI, USA, cat #B7) or goat anti-NeuroD (1:25000; Santa Cruz Biotechnology, CA, USA, cat #sc-1084). The next steps included a series of sequential incubations, first with the secondary antibody (biotinylated anti-rabbit or anti-goat, 1:1000 respectively, Vector Laboratories, CA, USA, cat #BA-1000 and BA-5000 respectively), followed by the Avidin/Biotin complex (Vectastain Elite ABC kit; Vector Laboratories, cat #PK-6100), and the chromogen 3,3’-diaminobenzidine (DAB; Merck, cat #D8001) for signal detection (for NeuroD with nickel chloride; Merck, cat #339350). Tissue for Ki-67 quantification was counterstained with cresyl violet (Thermo Fisher Scientific, cat #405760100). Finally, sections were dehydrated in graded alcohols, immersed in xylene (Sharlab, Barcelona, Spain, cat #XI0052) and cover-slipped with Permount® (Thermo Fisher Scientific, cat #SP15-500). Positive cells were quantified in the dentate gryus of 8 sections/rat by an experimenter blind to the treatment groups with a Leica DMR light microscope (63 × objective lens). The total number of positive cells is represented in relation to the % number of cells present in control-male rats.

### Western blot

Hippocampal cell fate markers were evaluated by Western blot analysis. To do so, total homogenates were prepared from the right hippocampus as previously described in detail before (see [28, 29]). Brain proteins (40 μg; protein amount assessed by BCA, Thermo Fisher Scientific, cat #23225) were separated by electrophoresis on 10–15% SDS-PAGE minigels (Bio-Rad Laboratories, Hercules, CA, USA) and transferred (110 V, during 2 h 30 min) to nitrocellulose membranes that were incubated with appropriate primary antibody whose vendors and dilution conditions were the following: (1) Santa Cruz Biotechnology (CA, USA): anti-FADD (H-181) (1:2500; cat #sc-5559); (2) BD Biosciences (CA, USA): anti-Cyt c (1:5000; cat #556433); (3) Lab Vision Corporation (CA, USA): anti-Cdk5 (DC17) (1:1000; cat #DC17); and (4) Sigma-Aldrich (MO, USA): anti-NF-L (N5139) (1:1000; cat #5139NR4), anti-β-actin (clone AC-15) (1:10000; cat #A1978). Following incubation with the appropriate secondary antibody (anti-rabbit or -mouse IgG linked to horseradish peroxidase; 1:5000; Cell Signaling; cat #7074 and 7076, respectively), the immunoreactivity of selected proteins was detected by ECL reagents (Amersham, Buckinghamshire, UK) and signal of bound antibody was visualized with autoradiographic films (Amersham ECL Hyperfilm). Each band of interest was quantified by densitometric scanning (GS-800 Imaging Calibrated Densitometer, Bio-Rad), and percent changes in immunoreactivity for each marker were calculated for each rat with respect to control-male samples (100%) in various gels (each sample was run at least 2–3 times in different gels), and the mean value was used as a final estimate. Data were not normalized to any protein, since β-actin analysis served as a loading control (i.e., its content was not altered by any treatment conditions).

### Data statistical analyses

GraphPad Prism, Version 9.5 (GraphPad Software, USA) was used to analyze and plot all graphs following the guidelines in experimental pharmacology for displaying data and statistical methods [40]. Results are reported as mean values ± standard error of the mean (SEM), and individual symbols for each rat are shown within bar graphs. Parametric tests were used for statistical comparisons, since assumptions for normality of data distribution and homogeneity of variance (in case of analysis of variance) were met (i.e., D’Agostino-Pearson normality test). Three-way repeated-measure (RM) ANOVAs were performed when analyzing potential changes in body weight (g), total fluid (sum of liquid consumed from both bottles independently of its contents and expressed in ml) and water (volume consumed from a single water bottle through the course of the experimental procedures and expressed in ml). The independent variables of study were Sex, Bottle Choice (Ethanol vs. Water) and Time of Analysis (Day or Week). When measuring ethanol preference (%) and ethanol dose consumed (g/kg) data were analyzed with two-way RM ANOVAs (independent variables: Sex and Time of Analysis), since all rats were exposed to the choice of one ethanol bottle. Cumulative ethanol load was compared between male and female rats with an unpaired two-tailed *t*-test. Finally, the regulation of hippocampal markers of induced neurotoxicity (i.e., neural progenitors and/or cell fate markers) was performed with a two-way ANOVA (independent variables: Sex and Experimental Group). Individual values were normalized to control male rats, to estimate the % magnitude of change. Tukey's or Sidak's multiple comparisons tests were performed for *post hoc* pair-wise statistical comparisons when appropriate. The level of significance was set at *p* ≤ 0.05. Interactions among variables were only reported when relevant and/or significant.

## Results

All datasets generated during and/or analyzed during the current study are available from the corresponding author on reasonable request. Moreover, a table with full statistical analysis is included as Supplementary Materials.

### No changes in body weight by ethanol voluntary consumption

The impact of voluntary ethanol consumption on weekly body weight was analyzed (see Supplementary Table S1). While there was no effect of Bottle of Choice (*F*_1,38_ = 0.16, *p* = 0.692), the expected significant effects of Sex (*F*_1,38_ = 281.8, ###*p* < 0.001; Fig. 1b) and Time of Analysis (*F*_6,228_ = 323.6, *p* < 0.001) were observed (i.e., increased weight in male vs. female rats, and progressive body weight gain over time during adulthood).

### Intermittent access to 20% ethanol in a two-bottle choice test for 3 consecutive days

**Fig. 2:**
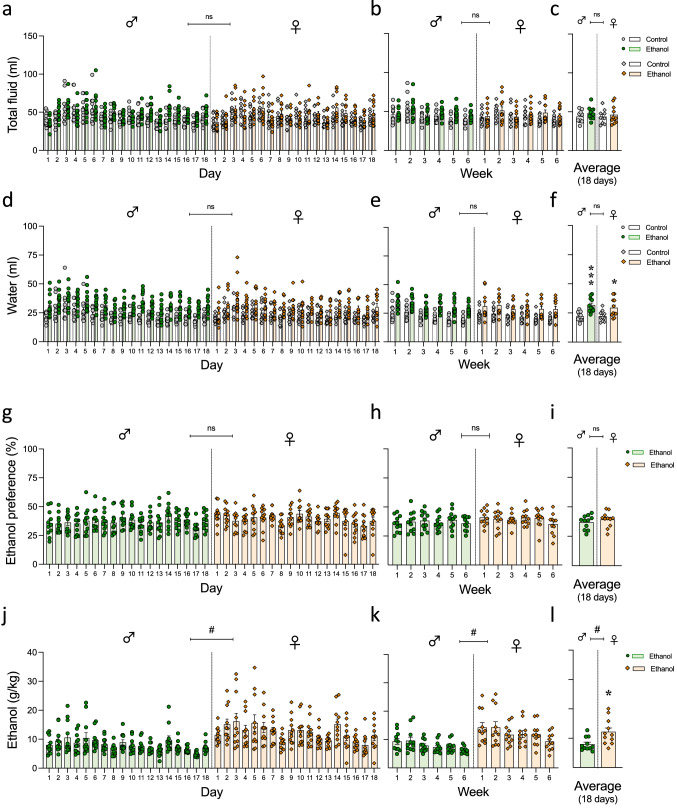
Intermittent access to 20% ethanol in a two-bottle choice test for 3 consecutive days. **a–c** Total fluid (ml) and **d–f** Water (ml) intake daily **(a–d)**, weekly (**b–e)** and expressed as the 18 days overall average (**c–f)**. Groups of treatment: Control-male (*n* = 10); Ethanol-male (*n* = 11); Control-female (*n* = 10); Ethanol-female (*n* = 11). Columns represent mean ± SEM of the amount of liquid consumed in ml. Individual symbols are shown for each rat. Three-way RM ANOVAs showed no significant effects of Bottle Choice or Sex. **g–i** Ethanol preference (%) and **j–l** Ethanol (g/kg) consumption daily (**g–j)**, weekly (**h–k)** and expressed as the 18 days overall average (**i–l)**. Groups of treatment: Ethanol-male (*n* = 11); Ethanol-female (*n* = 11). Columns represent mean ± SEM of the preference for ethanol (expressed as a % value) or ethanol dose consumed (g/kg). Individual symbols are shown for each rat. #*p* < 0.05 when comparing the general effect of sex (two-way repeated-measures ANOVA); *p < 0.05 when comparing the dose consumed by female rats vs. male rats (Student’s *t*-test)

When evaluating how the procedure might have affected daily total fluid intake (ml) (a total of 18 days; 3 days/week), the results showed that there were no significant effects of Bottle Choice (*F*_1,38_ = 1.48, *p* = 0.231) or Sex (*F*_1,38_ = 0.14, *p* = 0.709), but a significant effect of Time of Analysis (*F*_17,646_ = 25.63, *p* < 0.001) (see Fig. 2a and Supplementary Table S1); results that could also be represented as the average 3-day consumption in a given week (see Fig. 2b). When calculating the average daily intake across all 18 days, male rats consumed an average of 45.9 ml of daily fluid, while female rats consumed 44.8 ml, and these total volumes were not affected by the fact that rats had or not access to ethanol (Fig. 2c and Supplementary Table S1). However, for water intake, the results showed a significant effect of Bottle Choice (*F*_1,38_ = 19.84, *p* < 0.001) and Time of Analysis (*F*_17,646_ = 20.49, *p* < 0.001), but no effect of Sex (*F*_1,38_ = 0.35, *p* = 0.555) (see Fig. 2d); results that could also be represented as the average 3-day consumption in a given week (see Fig. 2e). Rats that had access to an ethanol bottle drank more water than those with no access (i.e., water intake was calculated as the average of both water bottles), as represented by the daily intake averaged across all 18 days (see statistics analysis in Supplementary Table S1). While male and female control rats consumed similar amounts of water (an average of 26.8 vs. 25.8 ml per day, respectively), rats with access to one bottle of ethanol drank, in average, more water every day (males: + 9.7 ml, ***p < 0.001; females: + 7.2 ml, *p = 0.023 vs. controls; Fig. 2f) as represented by the significant effect of Bottle of Choice (Supplementary Table S1).

When calculating ethanol preference (%) for rats exposed to both liquids, the results showed that there was no significant effect of Sex (*F*_1,20_ = 0.74, *p* = 0.400), but a significant effect of Time of Analysis (*F*_17,340_ = 3.54, *p* < 0.001) (see Fig. 2g and Supplementary Table S1). The fluctuations observed across days dissipated when results were represented as the average 3-days consumption in a given week (*F*_5,100_ = 2.02, *p* = 0.083; see Fig. 2h, Supplementary Table S1). In general, no sex differences were observed in ethanol preference, with male rats showing an average preference of 37.0% while females showed a preference of 39.2% (Fig. 2i). Although the preference for ethanol was the same for both sexes, when evaluating the dose of ethanol consumed (g/kg), a significant effect of Sex displayed in the statistical analysis (*F*_1,20_ = 7.82 #*p* = 0.011; Fig. 2j), together with an effect of Time of Analysis (*F*_17,340_ = 13.07, *p* < 0.001); results also detected when the average 3-day consumption in a given week was represented (see Fig. 2k, Supplementary Table S1). Female rats showed a higher dose of ethanol consumed (12.2 g/kg) when compared to male rats (8.0 g/kg), with a mean increase of 4.2 g/kg (*p = 0.011 vs. male rats; Fig. 2l, Supplementary Table S1). Interestingly, when calculating the cumulative ethanol load across all experimental days, female rats consumed a total of 219.2 g/kg as compared to males that consumed only 143.8 g/kg (*t* = 2.80, *df* = 20, *p* = 0.011; data not shown in figures).

### Decreased survival of neural progenitors by ethanol voluntary consumption as a sign of ethanol-induced neurotoxicity

**Fig. 3:**
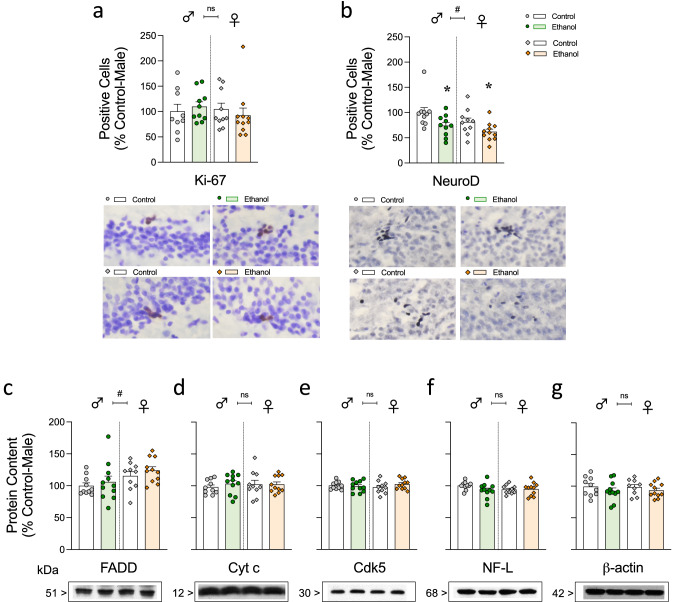
Evaluating signs of ethanol-induced neurotoxicity in hippocampus. Quantitative analysis of **a** Ki-67 and **b** NeuroD + cells in the left dentate gyrus of the hippocampus by immunohistochemistry or of **c** FADD**, d** Cytochrome c (Cyt c)** e** Cdk5,** f** NF-L and **g** β-actin protein content by western blot analysis in the hippocampus of male and female rats exposed to the two-bottle choice test (Control vs. Ethanol) on D38. Groups of treatment: Control-male (*n* = 10); Ethanol-male (*n* = 11); Control-female (*n* = 10); Ethanol-female (*n* = 11). Columns represent mean ± SEM of the number of + cells quantified in 8 sections from the middle part of the hippocampus and expressed as % change vs. control-male rats or of n experiments per group and expressed as a percentage of Control-male-treated rats. Individual symbols are shown for each rat. Two-way ANOVAs evaluating the potential effects of Bottle Choice (ethanol vs. water) and Sex. #*p* < 0.05 when comparing the general effect of sex and **p < 0.01 when comparing the effects of having access to a bottle with ethanol vs. control (general effect independently of sex). Bottom panels: representative images showing individual Ki-67 (brown labeling in the blue granular layer) and NeuroD (dark blue labeling in the blue granular layer) cells taken with a light microscope (40 × objective lens) or representative immunoblots depicting labeling of FADD, Cyt c, Cdk5, NF-L and β-actin are shown for each set of experiments. Other representative images for Ki-67 and NeuroD labeling and/or full immunoblots from which images were taken could be found in Supplementary Materials

The number of Ki-67 + cells was used to measure potential changes in cell proliferation for all groups as compared to control-male rats and expressed as % of its mean value (700 ± 102 Ki-67 + cells; expected number of cells for male rats of this age [37, 39]). The results showed no significant effects of Bottle Choice (*F*_1,36_ = 0.01, *p* = 0.925) or Sex (*F*_1,36_ = 0.24, *p* = 0.627) (see Fig. 3a, and Supplementary Table S1). As for NeuroD, the mean number of + cells in control-male rats (used to calculate the % changes for the rest of the groups) were 6616 ± 246 NeuroD + cells, also in line with prior data in male rats of this age [37, 39]. In this case, ethanol access decreased the survival of neural progenitors, as measured through the number of NeuroD + cells, and as observed by the significant effect of Bottle Choice (*F*_1,37_ = 8.98, **p = 0.005); rats with access to ethanol displayed an average of 22.7% lower NeuroD + cells when compared to control rats, independently of sex (Fig. 3b, Supplementary Table S1). This effect was observed for each sex when analyzed separately (male rats: -26%, *p = 0.042; female rats: -19%, *p = 0.05; see Fig. 3b, Supplementary Table S1). Moreover, female rats showed lower NeuroD + cells (a general drop of 15.5%) than male rats as observed by the significant effect of Sex (*F*_1,37_ = 4.19, #*p* = 0.048) (Fig. 3b). Correlation analysis were performed between the different parameters of ethanol consumption (i.e., average intake, preference and even with cumulative intake across all days) and the number of hippocampal NeuroD + cells; however, none of these variables were predictive of the later changes observed (data not shown).

Besides, no other signs of neurotoxicity were induced by ethanol voluntary consumption during adulthood when measured through several key cell fate markers (i.e., FADD, Cyt c, Cdk5, NF-L) by western blot analysis at the specific time-point of consideration (see Fig. 3c-f, Supplementary Table S1). The only significant change observed through the two-way ANOVAs performed was the significant effect of sex for FADD (*F*_1,38_ = 5.84, #*p* = 0.021; + 16.7% more FADD content for female adult rats independently of Bottle Choice; see Fig. 3c). β-actin was quantitated and used as a loading control as it was not altered by any treatment conditions (see Fig. 3g).

## Discussion

The main results showed moderate signs of ethanol neurotoxicity in hippocampus at the level of decreased neuronal progenitors (NeuroD + cells), effects that were independent of sex. Overall, the present results suggested that even in a situation where no escalation of ethanol intake occurred across time (a pattern mimicking a potential recreational use of ethanol in people), certain signs of neurotoxicity emerged, suggesting that sporadic ethanol use could still induce certain consequences in adult rats.

Our results aligned with several prior studies reporting that adult female rats, when given access to ethanol, consumed more (in g/kg), than their male counterparts (e.g., [12–14, 35],). In fact, the absolute volume of ethanol consumption did not different between sexes, however, given the smaller body weight of adult females, their total consumption in a g/kg basis was higher (around 4.2 g/kg higher per day in average). However, in comparison with other intermittent paradigms of ethanol accessibility (i.e., 24-h periods of access on alternate days of the week), the schedule followed in the present study (ethanol access continuously for 3 days a week followed by 4 days of withdrawal during 6 weeks) did not lead to a temporal increase in intake, neither for male nor for female rats. In our particular conditions, the range of intake (lowest and highest value) among individual rats from each group fluctuated between 5.5 and 12.8 g/kg (mean value of 8.0 g/kg) for adult males, and between 6.6 and 20.1 g/kg (mean value of 12.2 g/kg) for adult female rats, which are considered moderate to large doses of ethanol (reviewed by [4]). This differs from prior data describing that Sprague-Dawley rats consume low to moderate levels of ethanol and achieve lower ethanol preference rates than other strains (reviewed by [5]). That seems to be one of the reasons why the majority of studies reported in the literature were done in Long-Evan or Wistar rats, although escalation in ethanol intake has been reported for all strains, including Sprague-Dawley (reviewed by [5]). Moreover, many studies have revealed that not only strain, but also many other factors could affect the behavioral results, such as age, sex and particular environmental conditions (e.g., number of available bottles, ethanol concentration and temporal accessibility, etc.; reviewed by [41]), and could justify the lack of escalation in ethanol intake (g/kg) and/or in ethanol preference observed with the current paradigm.

In terms of ethanol preference across time, our results reported no significant differences by sex, with male rats showing an average preference of 37.0% while females showed a preference of 39.2%. The preference was below 50% since rats with access to ethanol drank more water than ethanol, although the total amount of fluid intake did not change when compared to their respective control group (i.e., comparing rats exposed to two-water bottles vs. rats given unlimited access to ethanol). Still, these levels of preference seemed comparably higher to others previously reported in the literature showing ethanol preferences starting around 15% on day 1, and then escalating up to 35–40% following several weeks of exposure (e.g., [9]). Our results reported similar amounts of ethanol consumption every week (no drop in consumption observed with time), but no escalation in preference over time, suggesting no progression into an addictive-like phenotype. Contrary, we could argue that in our particular experimental conditions, and right from the start of the procedure (week 1 and onward), rats showed good preference levels for ethanol, in line with the high values of intake described above, and as compared to prior studies (reviewed by [4, 5]), and maybe representing steady celling values preventing further escalation. In fact, we propose that this pattern of consumption might be mimicking the recreational use of ethanol during weekends in people. Overall, the present paradigm did not show escalation of ethanol intake, but it reproduced high levels of consumption, mimicking a common paradigm of intake for most adult individuals, in which to evaluate potential signs of neurotoxicity induced by ethanol in hippocampus.

Overall, the neurochemical results showed a moderate impact of ethanol on the markers evaluated in hippocampus. In particular, both male and female rats exposed to ethanol intermittently during 6 weeks presented lower hippocampal NeuroD + cells, a sign of decreased survival rate of neural progenitors (immature neurons). These results align with prior studies reporting damaging effects of ethanol on several stages of adult neurogenesis, including neuronal progenitors (e.g., [21]; reviewed by [22]), to describe a similar impact but following a distinct scheduling and including both sexes. Contrarily to other studies that reported sex differences in the regulation of hippocampal neurogenesis following ethanol use (e.g., [19, 20]), the present data showed similar changes on NeuroD for both sexes, even though females consumed more ethanol (in g/kg). This was interesting given the basal differences observed in Neuro + cells, having female rats lower neuronal progenitors as compared to male rats. However, independent of these basal differences, ethanol exposure affected both sexes similarly. This goes along with the sex effects priory reported in the different stages of hippocampal neurogenesis in the literature [42, 43], and in particular with the reported slower hippocampal neuronal maturation in females compared to males [43, 44].

In line with these baseline sex disparities, adult female rats also showed higher hippocampal FADD protein content than males, suggesting differences in baseline hippocampal neurotoxicity rates in line with their, just reported, lower NeuroD levels. Interestingly, FADD and/or the rest of the potential markers evaluated (Cyt c, Cdk5, NF-L) showed no signs of induced toxicity by ethanol exposure that could accompany the observed moderate decreased number of neuronal progenitors, even though prior studies have paired decreases in NeuroD with increases in FADD content but following cocaine exposure [27]. Although no prior studies have evaluated hippocampal FADD regulation following ethanol consumption in vivo, indications of ethanol-induced apoptosis through Fas-mediated pathways were described in several in vitro lines of human liver adenocarcinoma cells [45, 46]. Considering the present results, probably the amount of ethanol exposure was not sufficient to induce changes in FADD protein. Alternatively, the expected initial acute change might have adapted following a 6-week repeated ethanol exposure. These explanations might also justify the lack of effects on the other markers evaluated in hippocampus by ethanol exposure. For example, no changes were observed in Cdk5 regulation, although prior studies suggested that ethanol exposure resulted in Cdk5 overactivation in cortex and cerebellum [47], as well as in increased levels of Cdk5 and its activator p25 in hippocampus. These prior experiments suggested that interfering with this pathway might serve as a potential therapeutic approach to prevent ethanol-induced neurotoxicity in the brain [48], and although our results did not find Cdk5 altered at the end of the procedure, maybe it still might be regulated during ethanol consumption. Similarly, we found no signs of neurotoxicity as measured by NF-L protein content in hippocampus, though it was found hyperphosphorylated in hippocampus in response to ethanol toxicity [32], and other drugs of abuse (i.e., opiates/opioids and MDMA) decreased its content, proposing NF-L as a marker of structural damage to particular brain regions [29, 49].

In conjunction, the present results demonstrated a moderate neurotoxic effect of ethanol exposure as observed by decreased NeuroD + cells in the dentate gyrus of male and female rats, without any other ethanol-evoked changes (i.e., number of Ki-67 + cells or protein contents of FADD, Cyt c, Cdk5, NF-L). The discrepancies with prior reports that suggested signs of neurotoxicity through the dysregulation of these markers by ethanol exposure could be due to different experimental conditions, including the paradigm of ethanol used, the specific time of evaluation, and/or whether experiments were performed in vitro or in vivo, among other factors. Therefore, our pool of data is limited by how the experimental procedures were designed and could only be discussed in that context, yet presented relevant results while including sex as a biological variable. Other complementary experiments, both behavioral and neurochemical, would be required to demonstrate other signs of neurotoxicity, besides our moderate changes, and thus our conclusions are limited to the current design which particularly studied the regulation of the proposed markers on a single time-point of analysis at the end of the experiment.

In conclusion, this study sheds light on some of the potential negative consequences induced by a common pattern of ethanol consumption during adulthood, with no apparent signs of increased intake and/or changes in ethanol preference, but with certain signs of moderate neurotoxicity. Overall, our data suggest that the consumption of ethanol during adulthood, although at a recreational level, could also lead to certain brain harm.

## Supplementary Information

Below is the link to the electronic supplementary material.

Supplementary file1 (PDF 3046 KB)
