# Supplementary material for: Evaluating signs of hippocampal neurotoxicity induced by a revisited paradigm of voluntary ethanol consumption in adult male and female Sprague-Dawley rats
Source: Pharmacol Rep. 2023 Feb 20;75(2):320–30. doi: 10.1007/s43440-023-00464-6 (PMC10060276; doi:10.1007/s43440-023-00464-6)

Supplementary Table S1

Three-way repeated-measures ANOVA: F (DFn, DFd), *p* value

	Sex	Bottle of Choice	Time of Analysis	Sex x Bottle x Time
**Fig. 1b. Body weight (g/Week)**	F(1,38)=281.8; *p* <0.001	F(1,38)=0.16; *p* =0.692	F(6,228)=323.6; *p*< 0.001	F(6,228)=1.23; *p* =0.291
**Fig. 2a. Total fluid (ml/Day)**	F(1,38)=0.14; *p* =0.701	F(1,38)=1.48; *p* =0.231	F(17,646)=25.63; *p*< 0.001	F(17,646)=0.92; *p*= 0.548
**Fig. 2b. Total fluid (ml/Week)**	F(1,38)=0.14; *p* =0.701	F(1,38)=1.48; *p* =0.231	F(5,190)=21.95; *p*< 0.001	F(5,190)=0.77; *p*= 0.576
**Fig. 2d. Water (ml/Day)**	F(1,38)=0.35; *p* =0.555	F(1,38)=19.84; *p* <0.001	F(17,646)=20.49; *p*< 0.001	F(17,646)=1.02; *p*= 0.437
**Fig. 2e. Water (ml/Week)**	F(1,38)=0.34; *p* =0.564	F(1,38)=21.11; *p* <0.001	F(5,190)=25.36; *p*< 0.001	F(5,190)=0.40; *p*= 0.851

Two-way repeated measures ANOVA: F (DFn, DFd), *p* value

	Sex	Bottle of Choice	Sex x Bottle
**Fig. 2c. Total fluid (ml/18 days)**	F(1,38)=0.14; *p* =0.707	F(1,38)=1.48; *p* =0.232	F(1,38)=0.12; *p* =0.730
**Fig. 2f. Water (ml/18 days)**	F(1,38)=0.33; *p* =0.568	F(1,38)=21.06; *p* <0.001	F(1,38)=0.46; *p* =0.501

Two-way repeated measures ANOVA: F (DFn, DFd), *p* value

	Sex	Time of Analysis	Sex x Time
**Fig. 2g. Ethanol preference (%/Day)**	F(1,20)=0.74; *p* =0.400	F(17,340)=3.548; *p* <0.001	F(17,340)=1.32; *p* =0.179
**Fig. 2h. Ethanol preference (%/Week)**	F(1,20)=0.74; *p* =0.400	F(5,100)=2.02; *p* =0.083	F(5,100)=1.38; *p* =0.239
**Fig. 2j. Ethanol (g/kg/Day)**	F(1,20)=7.82; *p* =0.011	F(17,340)=13.08; *p* <0.001	F(17,340)=0.87; *p* =0.610
**Fig. 2k. Ethanol (g/kg/Week)**	F(1,20)=7.84; *p* =0.011	F(5,100)=17.90; *p* <0.001	F(5,100)=0.84; *p* =0.526

Unpaired *t* -test: *t* , *df* , *p* value

**Fig. 2i. Ethanol preference (%/18 days)**	*t* =0.85, *df* =20, *p* =0.404
**Fig. 2l. Ethanol (g/kg/18 days)**	*t* =2.80 *df* =20, *p* =0.011

Two-way ANOVA: F (DFn, DFd), *p* value

	Sex	Experimental Group	Sex x Experimental Group
**Fig. 3a. Ki-67 +cells (% Control-Male)**	F(1,36)=0.24; *p* =0.627	F(1,36)=0.01; *p* =0.925	F(1,36)=0.72; *p* =0.402
**Fig. 3b. NeuroD +cells (% Control-Male)**	F(1,37)=4.19; *p* =0.048	F(1,37)=8.98; *p* =0.005	F(1,37)=0.19; *p* =0.664
**Fig. 3c. FADD (% Control-Male)**	F(1,38)=5.84; *p* =0.021	F(1,38)=1.12; *p* =0.297	F(1,38)=0.04; *p* =0.852
**Fig. 3d. Cytc (% Control-Male)**	F(1,38)=0.13; *p* =0.725	F(1,38)=0.27; *p* =0.606	F(1,38)=0.30; *p* =0.589
**Fig. 3e. Cdk5 (% Control-Male)**	F(1,38)=0.02; *p* =0.883	F(1,38)=0.54; *p* =0.466	F(1,38)=1.46; *p* =0.235
**Fig. 3f. NF-L (% Control-Male)**	F(1,38)=0.58; *p* =0.449	F(1,38)=1.34; *p* =0.255	F(1,38)=2.09; *p* =0.157
**Fig. 3g. Beta-actin (% Control-Male)**	F(1,37)=0.01; *p* =0.999	F(1,37)=1.76; *p* =0.192	F(1,37)=0.04; *p* =0.840

Unpaired *t* -test: *t* , *df* , *p* value

**Fig. 3b. NeuroD +cells (% Control-Male)**	*t* =2.20, *df* =18, *p* =0.042	Male rats
	t =2.03, *df* =19, *p* =0.05	Female rats

Fig. 3a. Ki-67

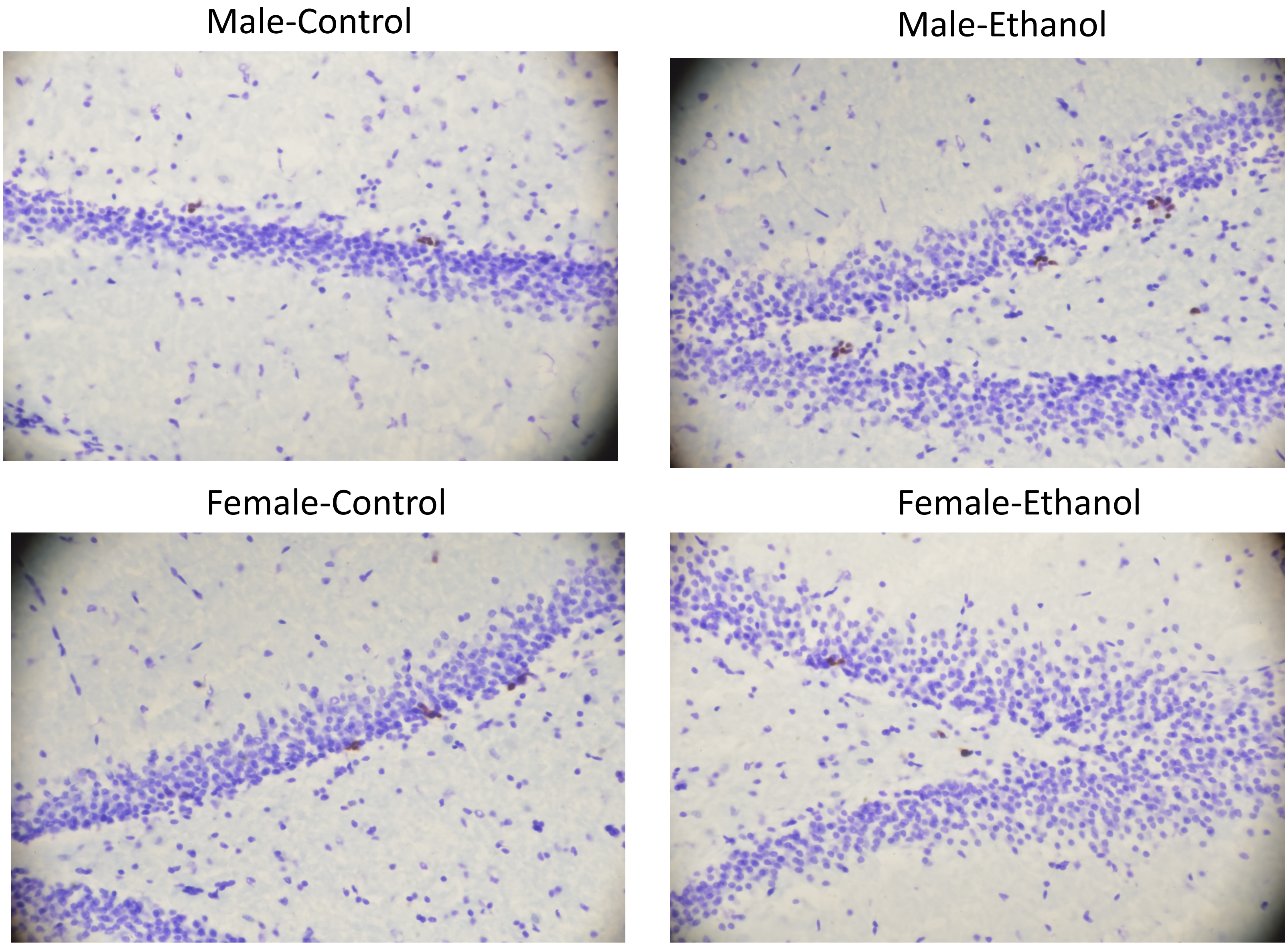

Fig. 3b. NeuroD

Male-Control

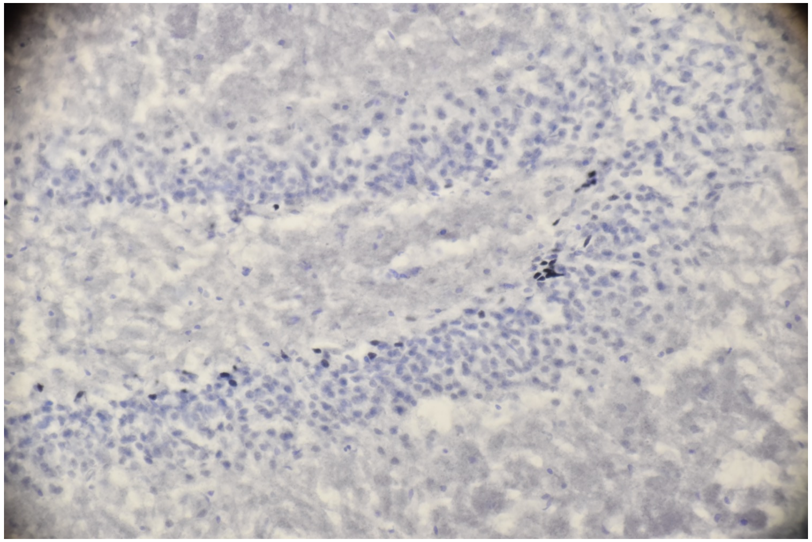

Male-Ethanol

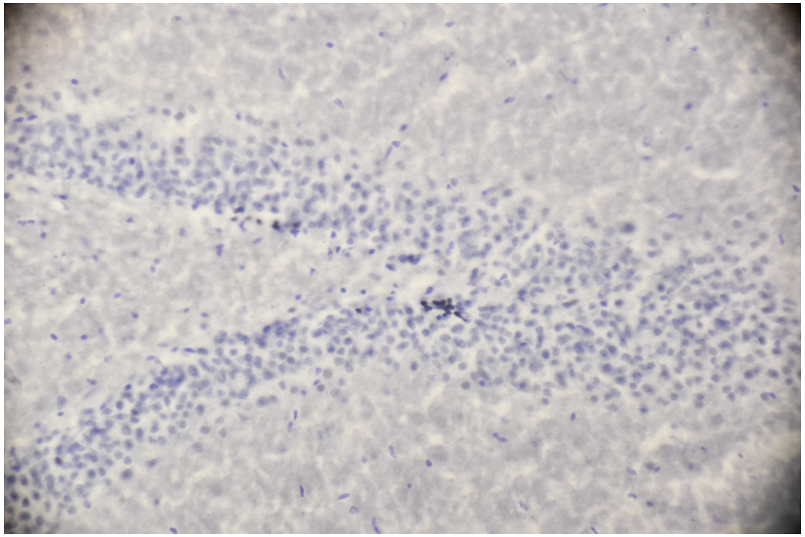

Female-Control

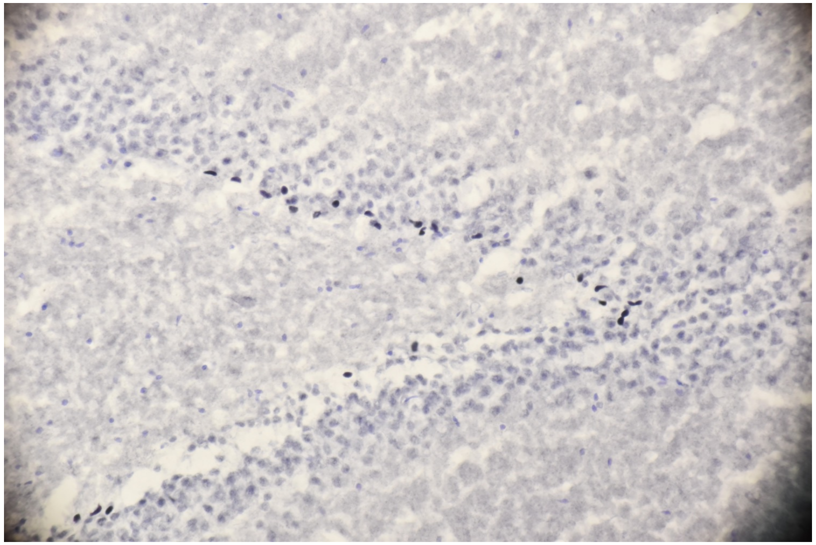

Female-Ethanol

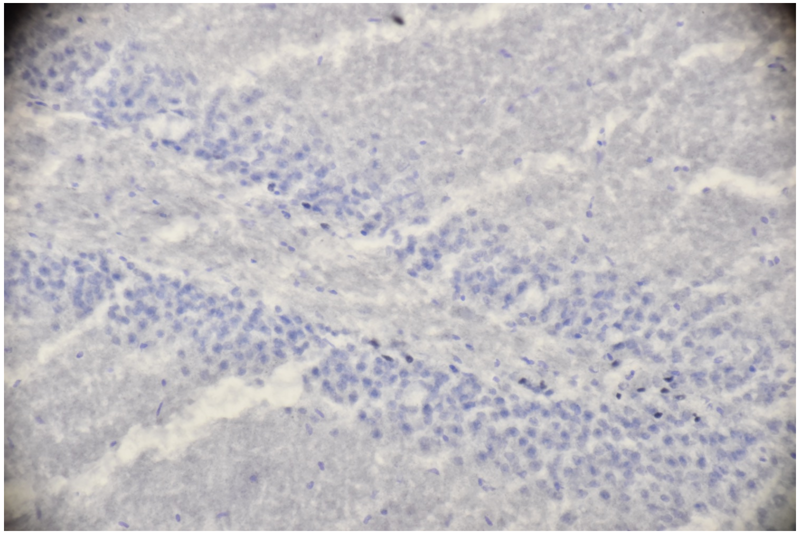

Extra images for NeuroD

Male-Control

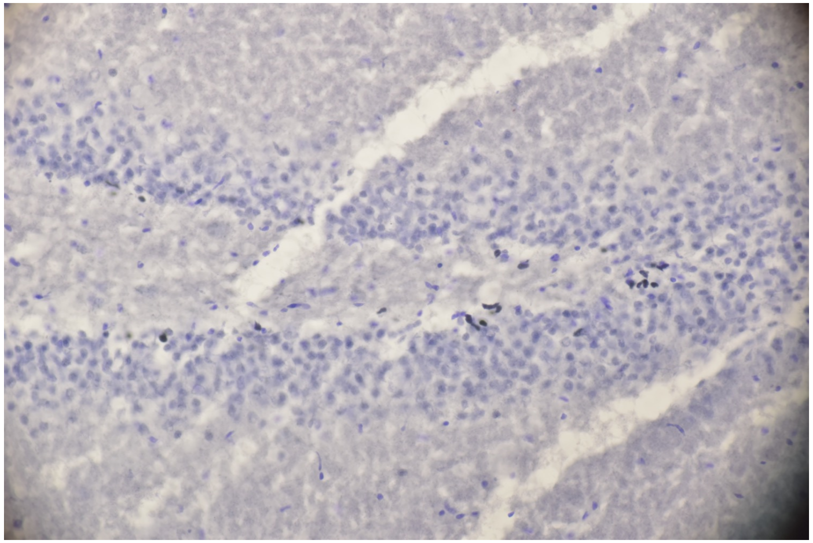

Male-Ethanol

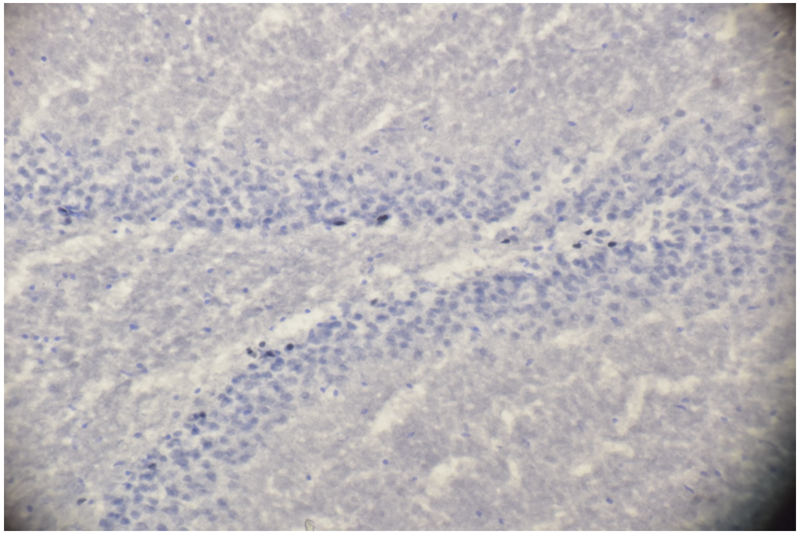

Female-Control

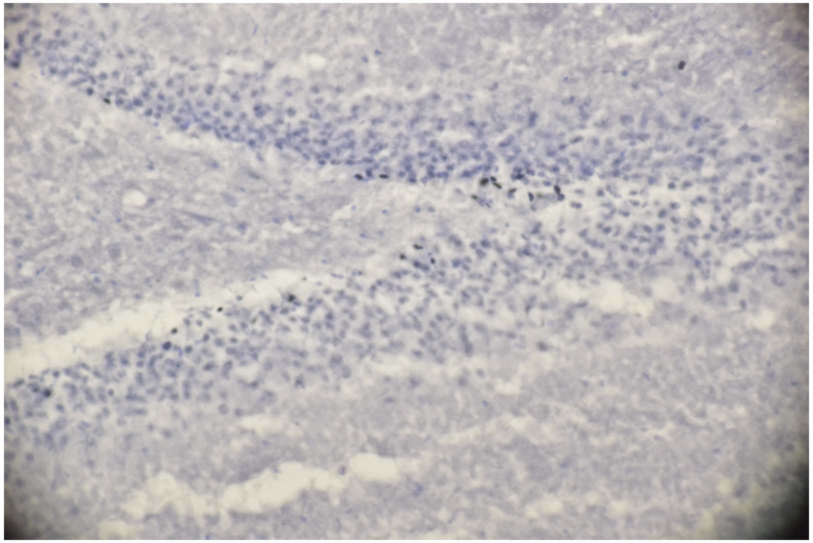

Female-Ethanol

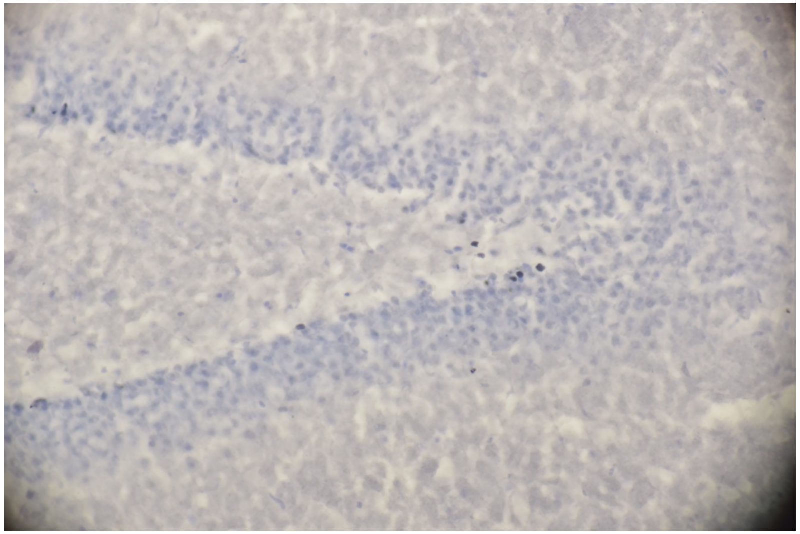

Fig. 3c: FADD

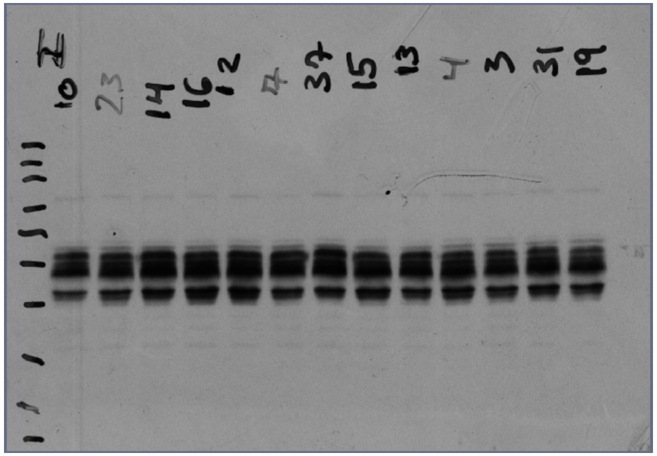

Fig. 3e: Cdk5

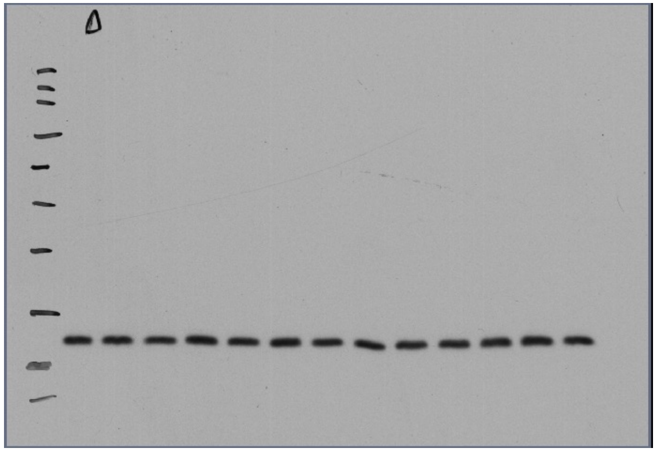

Fig. 3d: Cyt c

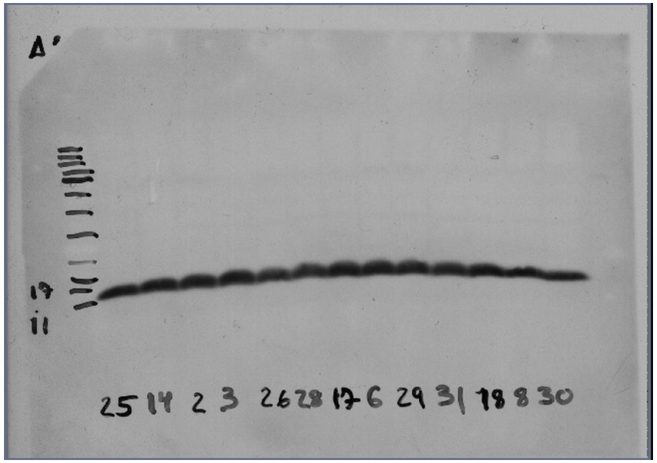

Fig. 3f: NF-L

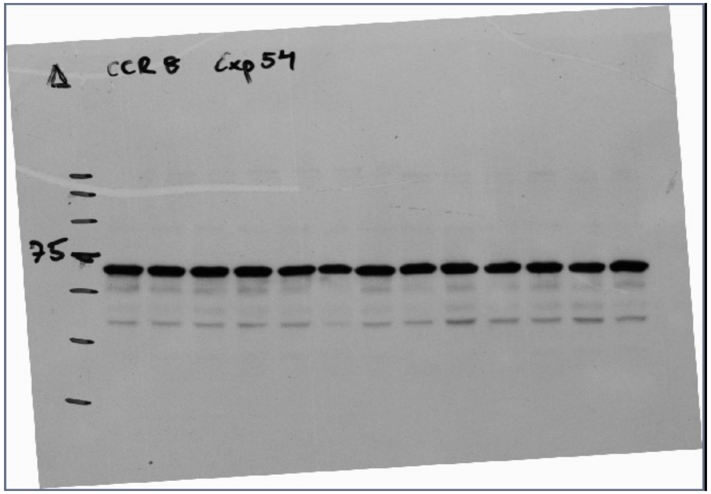

Fig. 3g: Beta-actin

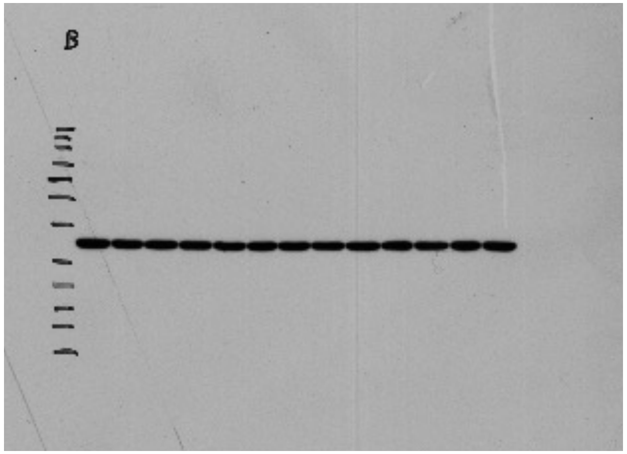

Supplement: Supplementary file 1 — Supplementary file1 (PDF 3046 KB) [file 43440_2023_464_MOESM1_ESM.pdf]
